# Comparing quality of life in lower extremity tumor patients undergoing limb salvage surgery and amputation: a meta-analysis

**DOI:** 10.3389/fonc.2023.1201202

**Published:** 2024-01-03

**Authors:** Nishant Banskota, Senlin Lei, Dechao Yuan, Xiang Fang, Sonali Banskota, Wenli Zhang, Hong Duan

**Affiliations:** ^1^ Department of Orthopedics, Orthopedic Research Institute, West China Hospital, Sichuan University, Chengdu, China; ^2^ Department of General Practice and Emergency Medicine, District Hospital Achham, Mangalsen, Sudurpaschim, Nepal

**Keywords:** limb salvage surgery, amputation, quality of life, SF-36, TESS, MSTS

## Abstract

**Purpose:**

Limb salvage surgery and amputation are two commonly performed procedures for lower extremity tumors. When comparing these procedures in tumor patients, it is important to consider their impact on quality of life (QOL) and functional mobility. These patients often experience physical, emotional, and psychological challenges, making these factors crucial in determining the most suitable treatment approach.

**Method:**

The outcomes of lower extremity tumors patients for QOL were collected from PubMed, MEDLINE, EMBASE, Cochrane, and Google Scholar until 28 February 2023. The physical function, mental health, role function, social function, emotional function, Toronto Extremity Salvage Score, and Musculoskeletal Tumor Society Score outcomes were analyzed to determine the differences between the two procedures.

**Results:**

Five articles were included according to the selection criteria with a total of 245 patients. The standard mean difference (SMD) values of each parameter were slightly higher in limb salvage surgery patients but not higher enough to produce statistically significant results; the SMD values for physical function and mental health were 0.72 and 0.04, respectively. This study did not report any heterogeneity or publication bias.

**Conclusions:**

QOL is a large and enhanced term, which carries its importance and is challenging to compare between any procedures. The minimal rise in SMD of different QOL parameters highlighted only a slight advantage of limb salvage surgery over amputation. Therefore, further research is required to explore the impact of this crucial topic.

## Introduction

The lower limb is associated with common malignant tumors such as osteosarcoma, Ewing tumor, and synovial cell sarcoma (1). Significant development has been made in the management of the lower extremity tumor in this modern era (2). Limb salvage surgery (LSS) has gained the popularity in managing the lower extremity tumors (3, 4). LSS has several advantages, but it is associated with a higher reoperation rate compared to amputation (5). Amputation is no longer the primary method of treatment for lower extremity tumors; however, there are still instances where it may be necessary, especially in advanced stages of the disease (6). The selection of treatment for tumor patients requires special consideration because of the aggressive nature of this lesion that may impact the daily life activities and long-term prognosis. In addition to that, as metastasis is more common in this malignant lesion, even with metastatic most surgeons’ attempts to preserve the limb, reserving amputation for tumors that cannot be managed with salvage surgery or palliative reasons (7).

A tumor in the lower extremity impacts a person’s daily life on functional, emotional, and psychological aspect. This has been stated in a systematic review studied by Bowman et al., in a cancer-related lower extremity lymphedema (8). Quality of life (QOL) is a large and enhanced term, due to its comprehensive nature, comparing QOL between different procedures is not an easy task. LSS not only has improved survival but also the appearance and the QOL (9, 10). Amputated patients’ QOL differs from person to person but is still perceived to have difficulty executing daily activities, which ultimately impact QOL in different aspects. On the contrary, in a study conducted by Malek et al., the authors found a similar QOL in amputee and limb-salvaged patients (11). The domains that are associated with and defined the QOL are not explained well but, in comprehensive physical function, psychological and social domains explain the majority of QOL (12). Not only the several studies produced contrasting results, numerous studies found better QOL in limb salvaged patients (13, 14) and other studies produced similar QOL in both these procedures (15, 16). In a study conducted by Sinha et al., amputee’s use of the prosthesis and comorbidities were found to be the most important factors influencing the physical function component of QOL, whereas employment status and comorbidities impacted mainly the mental health component of QOL (17).

Regardless of the surgery, patients treated for a bone tumor reported poorer QOL than expected from the normal population (18). The impact of amputation and LSS on daily life can be studied through different components, one important component being QOL. The recent advancements in the management of tumors have prolonged the survival of patients, and these surviving patients’ encounter with different problems in their life (19). In our study, SF-36 components were one of the major variables to be compared. The SF-36 has been increasingly reported in the published article and stated as a single variable that well defines QOL (20). The SF-36 measures eight scales: physical functioning, role physical, bodily pain, general health, vitality, social functioning, role emotional, and mental health (20). In this systematic review, we attempted to analyze all these parameters, not as a single variable as SF-36, to get a better understanding on the component of SF-36, which is more impacted. Along with these, two other major variables were TESS (Toronto Extremity Salvage Score) and MSTS (Musculoskeletal Tumor Society Score). Through searching more abundant lower extremity tumors literature, we conducted this meta-analysis to get a comprehensive conclusion in QOL differences among patients undergoing amputation and LSS. These results will help us to identify the exact differences in QOL components, especially the components that are largely impacted.

## Methods

This study was conducted following the guidelines of Preferred Reporting Items for Systematic reviews and Meta-Analyses (PRISMA) (21). The PRISMA chart is presented in the **Supplementary File**.

### Literature search

The relevant literatures and outcomes were searched through PUBMED, MEDLINE, Cochrane, EMBASE, and Google Scholar databases until 28 February 2023. The reference articles of relevant studies were also searched on different databases. Searches were confined to newer and recent studies, but due to the lack of the recent studies, search duration was expanded to include reliable and valid studies. The literature search criteria included studies with similar study periods and similar age groups to avoid any flaws and bias. Keywords used for searching literature included lower extremity tumors, LSS, amputation, QOL, MSTS, TESS, SF-36, and lower limb function. Literatures involving comparative study of LSS and amputation, impacting in daily life, performed for diseases other than tumors were also screened for getting better conclusive opinion for our study.

### Included studies

#### Inclusion criteria

1. Published studies focusing on patients diagnosed with musculoskeletal tumors in lower extremities.
2. Comparative studies focusing on QOL between LSS and amputation for lower extremities tumor.
3. Studies that had compared QOL components SF-36, TESS, and MSTS as their comparing parameters in lower extremity tumor patients undergoing LSS and amputation.

#### Exclusion criteria

1. Unpublished studies
2. Non-comparative studies in patients undergoing LSS and amputation for lower extremity tumors
3. Case reports, reviews, and letters to editors
4. Comparative study between LSS and amputation that lacked related clinical data

Conflicts and uncertainties regarding eligibility and viability of the data were resolved through discussions among the reviewers, when necessary. For the missing data in the respective studies, authors were contacted through the mail to retrieve the missing data.

### Data extraction and study selection

To determine whether the studies addressed the issues raised by our research, two authors of our study scanned all the abstracts and titles and collected the outcomes from the articles. After creating a structured table, the authors assembled all the data and associated information in a database. The following data were extracted from articles according to the inclusion criteria: the name of the first author, year of publication, study design and protocol, number of patients in each group, patients’ age and gender, SF-36, MSTS, and TESS.

### Quality assessment and outcome measurement

The studies included were both prospective and retrospective and covered similar study topics in the literature. Due to the similarities in the studies’ inclusion criteria, surgical techniques, and research periods, there was little to no bias in all of them. The Newcastle Ottawa Scale was performed for the quality assessment of this study (19). In our study, SF-36 components were set as a primary outcome, and the secondary outcomes were listed as TESS and MSTS. SF-36 constitutes eight components that all relate to the QOL in both physical and psychological aspects.

### Statistical analysis

The outcomes that were used in our study were SF-36 (which constitutes eight scales: physical functioning, role physical, bodily pain, general health, vitality, social functioning, role emotional, and mental health), MSTS, and TESS. All these comparing variables were continuous data. We used the software of the Cochrane Collaboration (Review Manager 5.2) to calculate the standard mean difference (SMD) and 95% confidence intervals (CIs). *I*² tests were used to assess statistical heterogeneity among the included papers (22). For statistically significant heterogeneity, I² value was *considered* to be greater than 0.5 (22). Heterogeneity was defined as low, moderate, or high based on the *I*² value (<40%: low; 30%–60%: moderate; 50%–70%: substantial; >75%: high) (22). *I*² illustrates the percentage of the total variability in effect estimates among trials that is due to heterogeneity rather than chance (23). A random-effects model was selected for heterogeneous data; otherwise, a fixed-effect model was selected. The relationship between a study’s precision and effect size is depicted in a funnel plot (24). It is a scatter plot that compares sample size to the estimated treatment effects from individual studies (horizontal axis) (24). Publication bias was identified through funnel plots, which exhibited an intervention effect from the individual study against the respective standard error (23). Asymmetry in the funnel plot, measured using regression analysis, is an indication of publication bias and a symmetrical funnel plot suggests no publication bias (24).

## Results

### Study selection

**Figure 1:**
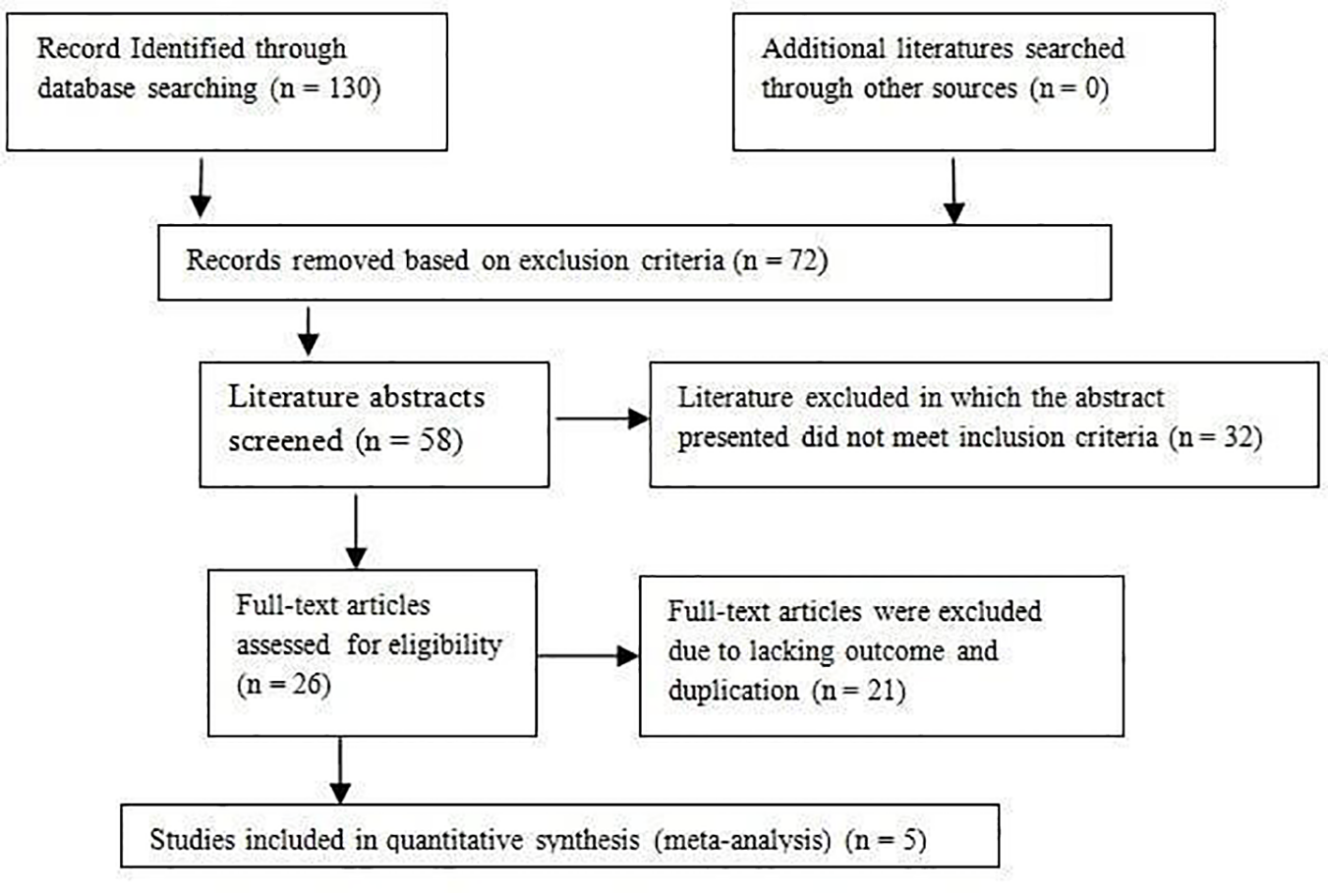
Flow chart of studies included and excluded.

In the study of the meta-analysis, 130 relevant articles were retrieved and 72 were excluded based on the exclusion criteria. The abstracts of the remaining 58 were screened, and 32 were excluded based on the exclusion criteria. After going through all the reviews of the remaining 26 studies, 10 were excluded due to lack of outcome related to our study (*n* = 10) and duplication in the study population with other articles (*n* = 11). Finally, a total of five articles were included in the meta-analysis. The study selection chart is presented in **Figure 1**. The characteristics and the information of the included studies are presented in **Table 1**, and the outcomes along with their mean values in respective studies are presented in **Table 2**.

**Table 1:** Characteristics of included studies.

Studies	Study period	Patient number	LSS	Amputation	Male/Female	Median age	Study design	Newcastle–Ottawa quality score	Country
Davis et al., 1999 (25)	1986–1995	36	24	12	NA	Amputation 34.4, LSS 30.4	Retrospective	8	Canada
Hingurange et al., 2003 (26)	1980–2000	62	47	15	40/26	Amputation 35, LSS 31	Retrospective	8	Germany
Vasquez et al., 2022 (27)	2002–2014	19	15	4	11/8	Amputation 20, LSS 21.5	Retrospective	9	Peru
Reijers et al., 2021 (28)	2008–2016	69	49	20	NA	Amputation 68, LSS 69	Retrospective	8	Netherlands
Ginsberg et al., 2007 (29)	2003–2005	59	41	18	NA	Amputation 20.13, LSS 20.13	Prospective	8	USA

NA (not available)—information not available in the respective trial.

**Table 2:** Outcomes of the included studies.

Reference	Physical function LSS/amputation	Mental health LSS/amputation	Role function LSS/amputation	Emotional function LSS/amputation	Social function LSS/amputation	TESS LSS/amputation	MSTS LSS/amputation
Davis et al., 1999 (25)	71.1(26)/45(28)	78.6(17)/70(28)	78.6(35)/47.5(46)	84.5(32)/86.7(32)	88.4(21)/78.8(21)	85.1(16)/74.5(19)	NA
Hingurange et al., 2003 (26)	91.6(6)/88.9(8)	88.3(21)/91.1(17)	65(30)/64.5(25)	72.7(22)/74(25)	77.7(25)/69.1(26)	NA	26.9(7)/22.8(5)
Vasquez et al., 2022 (27)	79.1(7)/78.04(5)	76.2(9)/71.6(7)	NA	NA	NA	NA	NA
Reijers et al., 2021 (28)	82.7(16)/62.7(23)	88.4(20)/90.8(19)	80.6(23)/67.5(31)	89.1(17)/89.2(19)	85.7(24)/85(19)	85.5(13)/72.2(21)	NA
Ginsberg et al., 2007 (29)	NA	NA	NA	NA	NA	86.4(9)/84.8(10)	21.8(4)/19.6(4)

NA (not available)—information not available in the respective trial; mean (SD) values reported for the respective.

Outcomes in the respective study.

### Outcome

There were a total of seven outcome included in our study in five studies; in the SF-36 components, three components: general health, bodily pain, and vitality were not included in our study due to the missing data.

### Physical function

**Figure 2:**
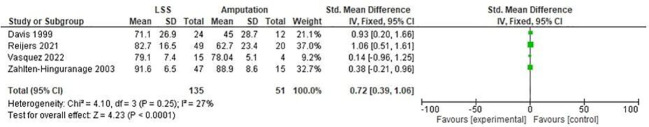
Forest plot of Physical function comparing in lower extremity tumor patients undergoing LSS and amputation.

In the included studies, four of the five studies reported physical function in both LSS and amputation patients. Fixed-model effects were used for the analysis of this outcome. Heterogeneity defining *I*² was 27%; hence, fixed-model effects were used for the analysis of this outcome. There was only a minimal difference in the SMD of physical function in lower extremity tumors patients treated with LSS when compared with amputation (SMD = 0.72, 95% CI [0.39, 1.06], Test for overall effect: *Z* = 4.23 (*P* < 0.0001). The forest plot for a physical function is shown in **Figure 2**.

### Mental health

**Figure 3:**
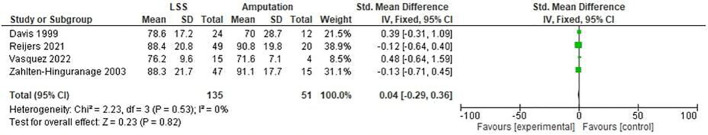
Forest plot of mental healthPhysical function comparing in lower extremity tumor patients undergoing LSS and amputation.

In the included studies, four of the five studies reported mental health in both LSS and amputation patients. A fixed-model effect was used for the analysis of this outcome. The SMD value of the LSS group when compared with the amputation group was only slightly higher (SMD = 0.04, 95% CI [−0.29, 0.36], test for overall effect: *Z* = 0.23 (*P* = 0.82). The forest plot for mental health is shown in **Figure 3**.

### Role function

**Figure 4:**
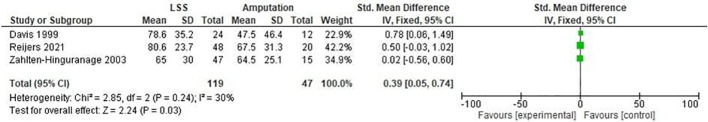
Forest plot of role function comparing in lower extremity tumor patients undergoing LSS and amputation.

In the included studies, three of the five studies reported role function in both LSS and amputation patients. A fixed-model effect was used for the analysis of this outcome. The SMD value of the LSS group when compared with the amputation group was only slightly higher (SMD = 0.39, 95% CI [0.05, 0.74], test for overall effect: *Z* = 2.24 (*P* = 0.03). The forest plot for the role function is shown in **Figure 4**.

### Emotional function

**Figure 5:**
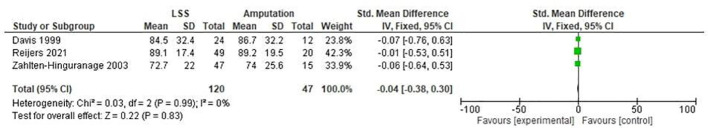
Forest plot of emotional function comparing in lower extremity tumor patients undergoing LSS and amputation.

In the included studies, three of the five studies reported emotional function in both LSS and amputation patients. A fixed-model effect was used for the analysis of this outcome. The SMD value of the LSS group when compared with the amputation group was only slightly lower (SMD = −0.04, 95% CI [−0.38, 0.30], Test for overall effect: *Z* = 0.22 (*P* = 0.83). The forest plot for the emotional function is shown in **Figure 5**.

### Social function

**Figure 6:**
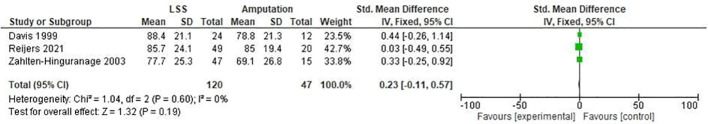
Forest plot of social function comparing in lower extremity tumor patients undergoing LSS and amputation.

In the included studies, three out of the five studies reported social function in both LSS and amputation patients. A fixed-model effect was used for the analysis of this outcome. The SMD value of the LSS group when compared with the amputation group was only slightly higher (SMD = 0.23, 95% CI [−0.11, 0.57], Test for overall effect: *Z* = 1.32 (*P* = 0.19). The forest plot for the social function is shown in **Figure 6**.

### TESS

**Figure 7:**
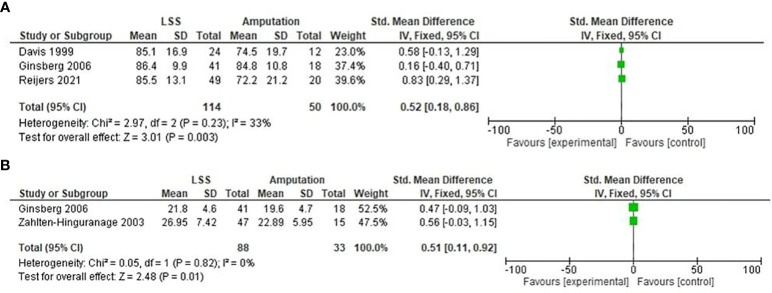
Forest plot of TESS **(A)** and MSTS **(B)** comparing in lower exremity tumor patients undergoing LSS and amputation.

In the included studies, three of the five studies reported TESS in both LSS and amputation patients. A fixed-model effect was used for the analysis of this outcome. The SMD value of the LSS group when compared with the amputation group was only slightly higher (SMD = 0.52, 95% CI [0.18, 0.86], test for overall effect: *Z* = 3.01 (*P* = 0.003). The forest plot for the TESS is shown in **Figure 7**.

### MSTS

In the included studies, two of the five studies reported MSTS in both LSS and amputation patients. A fixed-model effect was used for the analysis of this outcome. The SMD value of the LSS group when compared with the amputation group was only slightly higher (SMD = 0.51, 95% CI [0.11, 0.92], Test for overall effect: *Z* = 2.48 (*P* = 0.01). The forest plot for the TESS is shown in **Figure 7**.

### Sensitivity analysis

Sensitivity tests are performed to determine the reliability and validity of the results, which are conducted by removing each study in turn. The direction and magnitude of the combined estimates did not significantly alter when individual studies were excluded, demonstrating the validity of the meta-analysis’s findings and pointing to their significance and stability. Sensitivity analysis was performed for the two outcomes of our study physical function and mental health. The statistical value when the first study was excluded for physical function were OR = 0.67, 95% CI [0.29, 1.05], and *p* = 0.0005); when only the second study was excluded for physical function were OR = 0.53, 95% CI [0.11, 0.95], and *p* = 0.01; when only the third study was excluded for physical function were OR = 0.78, 95% CI [0.43, 1.13], and *p* < 0.0001; and when the fourth study was excluded for physical function were OR = 0.89, 95% CI [0.48, 1.30], and *p* < 0.0001. The statistical value when the first study was excluded for mental health was OR = −0.06, 95% CI [−0.42, 0.31], and *p* = 0.75); when only the second study was excluded for mental health were OR = 0.14, 95% CI [−0.28, 0.55], and *p* = 0.52; when only the third study was excluded for mental health were OR = −0.00, 95% CI [−0.34, 0.34], and *p* = 0.99; and when the fourth study was excluded for mental health were OR = 0.11, 95% CI [−0.28, 0.51], and *p* = 0.56. All the sensitivity analysis figures are presented in the **Supplementary File**.

### Publication bias

The funnel plot of the physical function, mental health, role function, emotional function, social function, TESS, and MSTS was shown in the figure added in the **Supplementary Materials**. The funnel plot is used for all the outcomes of our study. The results of the funnel plot suggested that there is no evidence of publication bias for any of the outcomes. All the funnel plot figures are added in the **Supplementary File**.

## Discussion

Since QOL has not always been defined or applied consistently, it has been challenging to compare the QOL results of different research directly (30). QOL meaning involves evaluating one’s overall life satisfaction with how they are currently operating in comparison to what they consider to be their ideal normal (31). QOL is acknowledged as a significant indicator of rehabilitation programs and has been primarily used to assess the efficacy of these programs or to compare individuals with amputations with the ill or healthy community (17, 32). The subscales, which reflect changes in physical health in the bone tumor patients, show that QOL values decline in individuals even with other different musculoskeletal disorders (33). SF-36 values listed by different studies are as whole SF-36 values. In this study, we tried to interpret the differences between each component in respective procedures. The physical and mental health components were the components reported by maximum studies, and in our study, there was only a slight difference in SMD 0.72 and 0.04, respectively, between these two procedures. In a prospective cohort study conducted by Kurozomi et al., in lower extremity tumor patients, the author found better QOL in LSS patients when compared to amputee patients (34). In this study, both physical function and mental health mean values were higher in the LSS group (34). Another study conducted by Eiser et al., in osteosarcoma and Ewing sarcoma patients in the lower limbs, resulted in similar QOL in LSS and amputation (18); these two studies’ results somehow were similar to our study.

These two procedures follow different entities: LSS patients follow the challenge of adjusting to the implants, meanwhile, amputee patients follow a different path (18). Amputation most of the time results from the failed LSS or prolonged infection or failed chemotherapy and radiotherapy treatments (18). Hence, the endurance for the amputee patients are more on the basis of prolonged duration of the treatments (18). The other reported components of SF-36 reported slightly higher SMD in LSS except in emotional function in which there was a negative SMD of −0.04. The QOL improvement in role function and social function has also been well established in a study conducted by Robert et al., in LSS patients undergoing surgery for lower extremity osteosarcoma (35). Patient’s inability to participate in life roles after treatment and their preoperative expectations can have a negative influence on the role and social aspect of QOL (36, 37). In a study conducted by Rosca et al., for analyzing the emotional aspects of amputee patients, the author found mixed emotions of sadness, depressive feeling, faith, strong desire, and positive hope (38).

The two functional quality parameters of our study TESS and MSTS values were slightly higher in LSS patients. This result can be justified by another study; in a study conducted by Yonemoto et al., the author discovered increased MSTS scores in high-grade osteosarcoma patients after undergoing LSS (39). The TESS score of our study could not be tallied with other literature due to the lack of publication in this related field. The TESS score was created with the goal of characterizing people at a specific moment and in the event of changes by tracking physical and functional impairment in everyday life (disability) (40). The TESS was created as an output of physical function (41). This consists of limitations in mobility, personal care, and in conducting regular daily life activities (41). Another functional evaluation parameter was MSTS in which higher scores are indicative of better functional mobility (42). The six components of MSTS are pain, function, emotional acceptance, lifting ability, manual dexterity, and hand positioning (43). This meta-analysis showed moreover similar results in both LSS and amputation patients and, when compared with the normal population, there was a huge difference. Hence, the management for this, after reviewing some literature, can be listed as early psychological consultation even before surgery for better acceptance of treatment, active family and group member talking, and regular counseling by the surgical team (44). In our study, two included studies were recently conducted and published in the Years 2021 and 2022 (27, 28), which highlights that these two treatment methods are in practice and established the validity of conducting this meta-analysis. Even though the study did not produce statistically significant result, the slight increase in SMD of physical and mental component in participants undergoing LSS might be encouraging for the authors to pursue for conducting research.

A few short comes of this meta-analysis should be explained. First, the insufficient and incomplete data from original studies made it difficult to adjust estimates by age, menopausal status, lifestyle, smoking, race, and so forth; more accurate analysis requires complete data and a bigger sample size. Second, we could not study other important components of SF-36 such as bodily pain, general health, and vitality. Third, we could not add other studies than retrospective and prospective studies such as RCT and non-RCT, as RCT and non-RCT studies are high-quality and could add more clarity on this rare topic; moreover, there is a lower chance of creating a bias for the readers. Fourth, since there were only limited studies published on this topic, it was hard to obtain statistically significant results. The systematic review is established on methods that are clearly defined to analyze, categorize, identify, and report aggregated evidence on a specific topic. The authors have followed a well-established protocol for conducting a systematic review, which includes a comprehensive search strategy, explicit inclusion and exclusion criteria, and a standardized form for data extraction and synthesis. The novelty of this meta-analysis is in the rarity of this topic as less research has been conducted on these topics; even though these two procedures are routinely performed on a regular basis, their impact on QOL has been rarely studied. We could only find meta-analysis in the lower extremity tumors undergoing these two procedures in osteosarcoma. The study was conducted without any flaw as analyzing patients in respective studies were moreover similar in age and study period; except for one study, all studies had adequate time duration for obtaining a reliable and valid result. Other beneficial points of this meta-analysis are listed below. First, a systematic review of the association between two surgical procedures (LSS and amputation) in lower extremity primary bone tumor patients is statistically more powerful than any single study. All the studies provided valid comparative data which analyzes the impacts of LSS and amputation on the QOL and functional mobility aspect. Second, all of the retrospective studies and one prospective study were of an excellent standard and confirmed our inclusion criteria. Third, even though the included studies were few, they still produced statistically significant results. Therefore, a conclusion has been established that highlights the need for further studies explaining every aspect of SF-36 and the steps, which could improve QOL in both pre- and post-operative phases of primary bone tumor patients.

## Data availability statement

The original contributions presented in the study are included in the article/**Supplementary Material**, further inquiries can be directed to the corresponding authors.

## Author contributions

Conception/design: WZ and HD; provision of study material: NB, and XF; collection and/or assembly of data: NB, SB, SL and DY; data analysis and interpretation: XF, SB, SL, and DY; manuscript writing: NB and SL; final approval of manuscript: WZ and HD; All the authors listed on the title page have read the manuscript, attest to the validity and legitimacy of the data and their interpretation, and agree to the manuscript’s submission to the Frontiers in Oncology: Surgical Oncology. All authors have read and agreed to the published version of the manuscript.
