# Supplementary material for: Comparing quality of life in lower extremity tumor patients undergoing limb salvage surgery and amputation: a meta-analysis
Source: Front Oncol. 2024 Jan 3;13:1201202. doi: 10.3389/fonc.2023.1201202 (PMC10792662; doi:10.3389/fonc.2023.1201202)
Supplement: Supplementary file 1 [file DataSheet_1.docx]

| **Supplementary Table 1: Newcastle– Ottawa Quality Assessment Scale for Cohort Studies** | | | | | | | | | | | | |
| --- | --- | --- | --- | --- | --- | --- | --- | --- | --- | --- | --- | --- |
| Authors | Represent-ativeness of the exposed cohort | Selection of the non-exposed cohort | Ascertain-ment of exposure | Demonst-ration that outcome not present at start | Compara-bility of cohort  (2 points) | | Assessment of Outcome | Follow-up length adequate for outcome to occur | Adequacy of Follow-up of cohorts (accounted for non-index hospitals) | Total Score  (9 points possible) | |  |
| Davis 1999 | 1 | 1 | 1 | 1 | 1 | 1 | | 1 | 1 | | 8 |  |
| Hingurange 2003 | 1 | 1 | 1 | 1 | 1 | 1 | | 1 | 1 | | 8 |  |
| Vasquez 2022 | 1 | 1 | 1 | 1 | 2 | 1 | | 1 | 1 | | 9 |  |
| Reijers 2021 | 1 | 1 | 1 | 1 | 1 | 1 | | 1 | 1 | | 8 |  |
| Ginsberg 2007 | 1 | 1 | 1 | 1 | 1 | 1 | | 1 | 1 | | 8 |  |

Supplementary figure 1: Funnel plot for publication bias

1. Physical function


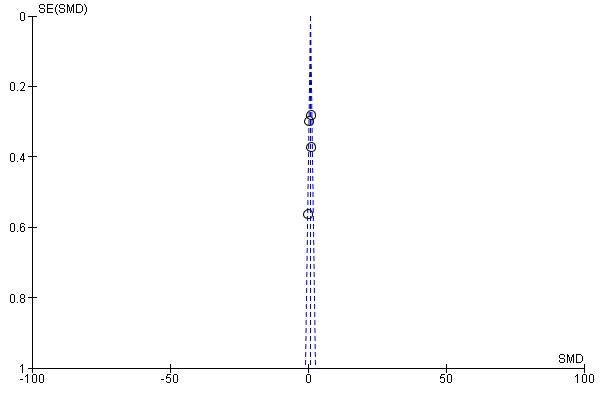


1. Mental health


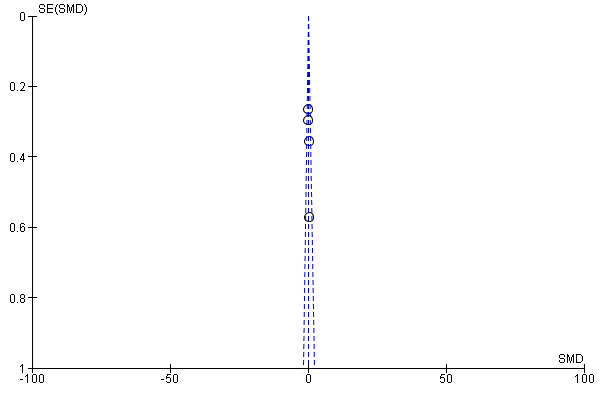


1. Role function


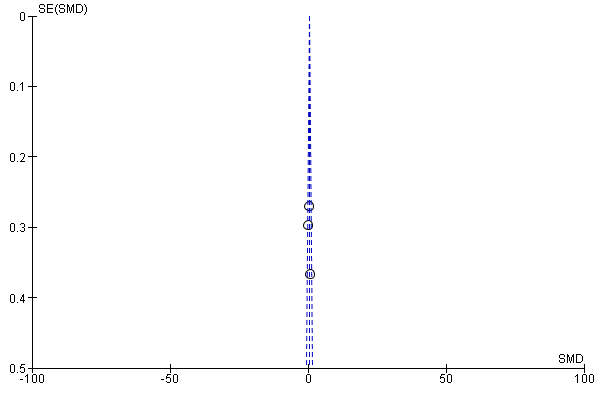


1. Emotional function


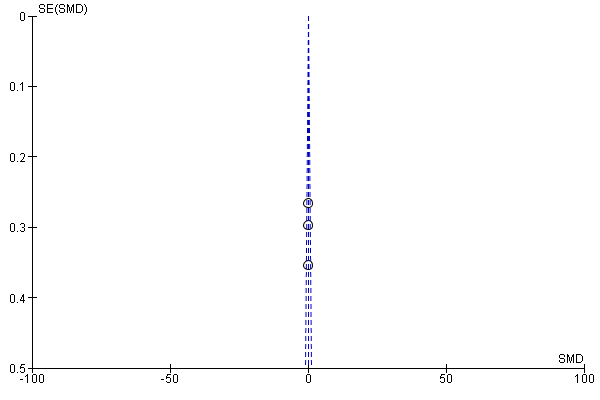


1. Social function


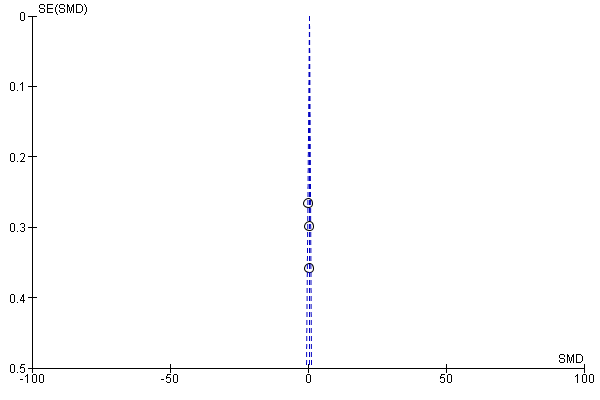


1. TESS


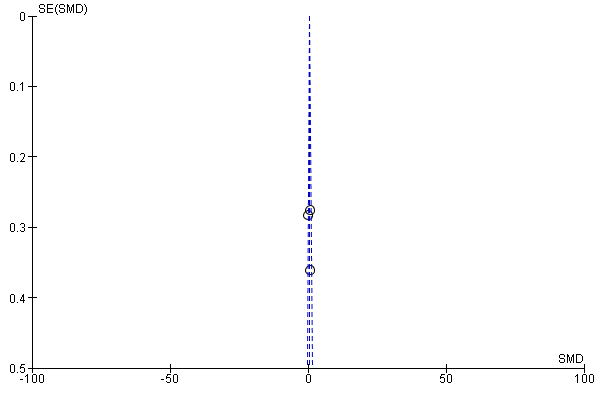


1. MSTS


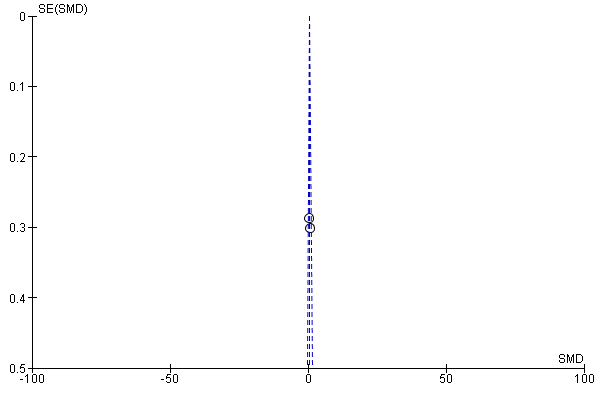


Supplementary figure 2: Results of leave-one-out sensitivity analysis


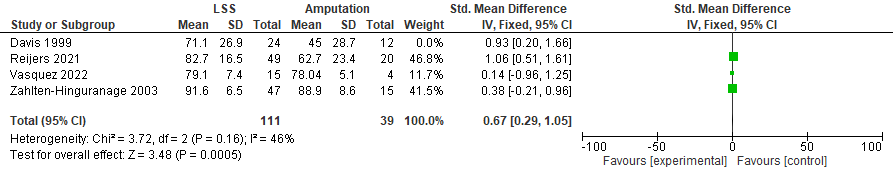


Supplementary Figure 2A: Forest plot for Physical function after excluding study by Davis et al.


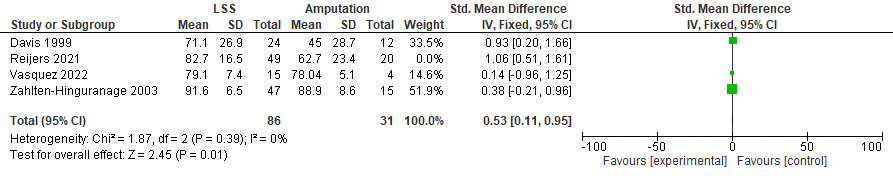


Supplementary Figure 2B: Forest plot for Physical function after excluding study by Reijers et al.


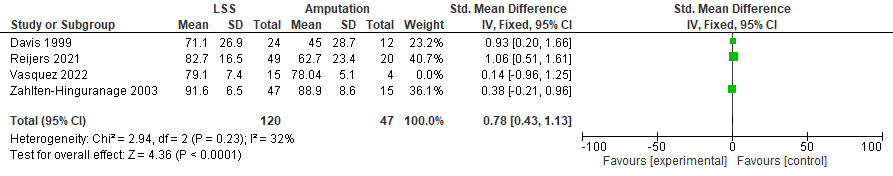


Supplementary Figure 2C: Forest plot for Physical function after excluding study by Vasquez et al.


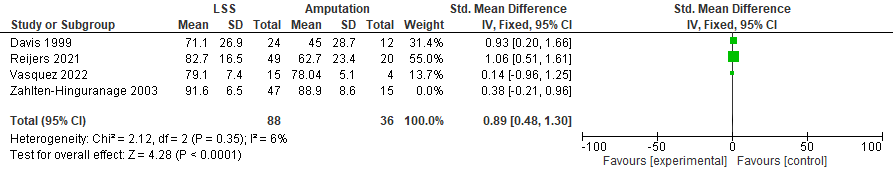


Supplementary Figure 2D: Forest plot for Physical function after excluding study by Zahlten-Hinguranage et al.


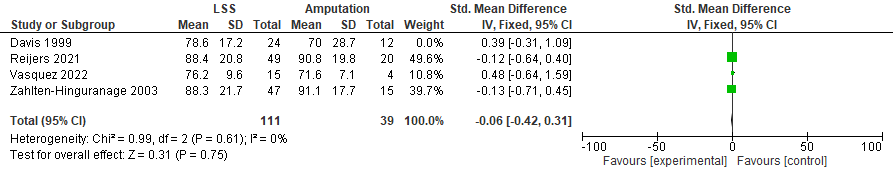


Supplementary Figure 2E: Forest plot for Mental health after excluding study by Davis et al.


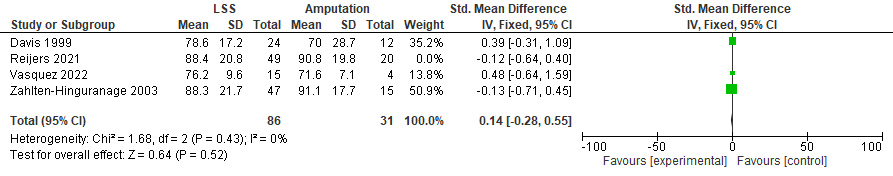


Supplementary Figure 2F: Forest plot for Mental health after excluding study by Reijers et al.


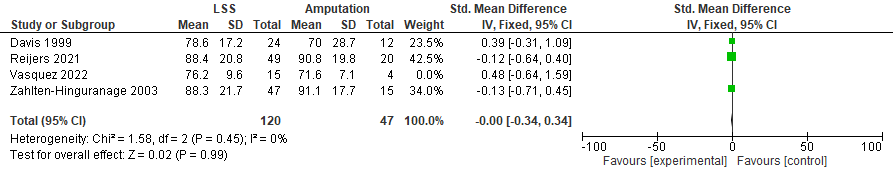


Supplementary Figure 2G: Forest plot for Mental health after excluding study by Vasquez et al.


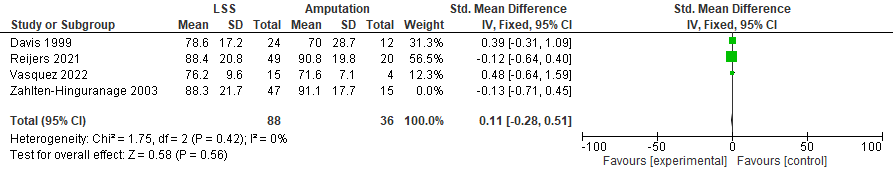


Supplementary Figure 2H: Forest plot for Mental health after excluding study by Zahlten-Hinguranage et al.
